# The interplay between PCOS pathology and diet on gut microbiota in a mouse model

**DOI:** 10.1080/19490976.2022.2085961

**Published:** 2022-07-04

**Authors:** Valentina Rodriguez Paris, Xin Yi Denise Wong, Samantha M Solon-Biet, Melissa C Edwards, Ali Aflatounian, Robert B Gilchrist, Stephen J Simpson, David J Handelsman, Nadeem O Kaakoush, Kirsty A Walters

**Affiliations:** a a Fertility & Research Centre, School of Clinical Medicine, University of New South Wales Sydney, Sydney, NSW, Australia; b Charles Perkins Centre, University of Sydney, Sydney, NSW, Australia; c ANZAC Research Institute, University of Sydney, Sydney, NSW, Australia; d School of Medical Sciences, University of New South Wales Sydney, Sydney, NSW, Australia

**Keywords:** Microbiome, hyperandrogenism, polycystic ovary syndrome, PCOS, diet, animal model

## Abstract

The gut microbiome has been implicated in polycystic ovary syndrome (PCOS) pathophysiology. PCOS is a disorder with reproductive, endocrine and metabolic irregularities, and several studies report that PCOS is associated with a decrease in microbial diversity and composition. Diet is an important regulator of the gut microbiome, as alterations in macronutrient composition impact the balance of gut microbial communities. This study investigated the interplay between macronutrient balance and PCOS on the gut microbiome of control and dihydrotestosterone (DHT)-induced PCOS-like mice exposed to diets that varied in protein (P), carbohydrate (C) and fat (F) content. The amount of dietary P, C and F consumed significantly altered alpha (α) and beta (β) diversity of the gut microbiota of control and PCOS-like mice. However, α-diversity between control and PCOS-like mice on the same diet did not differ significantly. In contrast, β-diversity was significantly altered by PCOS pathology. Further analysis identified an operational taxonomic unit (OTU) within *Bacteroides* (OTU3) with 99.2% similarity to *Bacteroides acidifaciens*, which is inversely associated with obesity, to be significantly decreased in PCOS-like mice. Additionally, this study investigated the role of the gut microbiome in the development of PCOS traits, whereby PCOS-like mice were transplanted with healthy fecal microbiota from control mice. Although the PCOS gut microbiome shifted toward that of control mice, PCOS traits were not ameliorated. Overall, these findings demonstrate that while diet exerts a stronger influence over gut microbiota diversity than PCOS pathology, overall gut microbiota composition is affected by PCOS pathology.

## Introduction

Polycystic ovary syndrome (PCOS) is the most frequently experienced endocrine disorder in women of reproductive age worldwide.^1–3^ The condition is characterized by reproductive, metabolic and endocrine abnormalities, including hyperandrogenism, ovulatory dysfunction, reduced fertility and a higher occurrence of obesity, insulin resistance, hepatic steatosis and dyslipidemia.^1,4^ Studies have examined different aspects of PCOS and varying interventions, however patient responsiveness to treatments vary widely^5^ . 30%–75% of women with PCOS display excess weight,^6,7^ and obesity aggravates PCOS symptoms.^8–10^ Weight loss improves PCOS features^6^ and the international evidence-based guidelines for the assessment and management of PCOS recommend lifestyle interventions, including diet and exercise, to be adopted by all women with PCOS.^11^

The gut microbiome has been implicated in the development of a range of metabolic disorders, including PCOS. Several studies in humans report a change in overall gut bacterial composition in women suffering from PCOS, with a decrease in the number of different species within a specific sample, alpha (α) diversity, and alterations in how similar one individual sample is to another, beta (β) diversity.^12–14^ However, others report no change in bacterial diversity in women with PCOS when compared to controls.^15^ The variations in results across studies may be due to small sample sizes and ethnic and PCOS phenotype differences, and hence, defined differences in gut microbiota communities and their precise role in PCOS pathogenesis remain unclear.

To understand in more detail the role of gut microbiota in PCOS, recent studies have employed the use of rodent models that display a breadth of PCOS-like characteristics.^16–23^ In the letrozole-induced PCOS rodent models, shifts of gut microbiota are observed.^16,20^ PCOS-like rats exhibited lower levels of *Lactobacillus, Ruminococcus* and *Clostridium* as well as higher levels of *Prevotella* compared to controls, which were restored to levels observed in controls by *Lactobacillus* transplantation or fecal microbiota transplantation (FMT) from healthy control rats.^16^ Importantly, *Lactobacillus* transplantation and FMT also resulted in restoration of cyclicity, improved ovarian follicle morphology, formation of corpora lutea and decreased testosterone and androstenedione levels.^16^ Taken together, these data suggest that modulation of gut microbiota may be a potential new treatment strategy for the management of PCOS. In a recent ground-breaking study, clear evidence of a causative role of gut microbiota in orchestrating PCOS traits comes from the finding that PCOS features can be induced in mice by FMT from women with PCOS.^24^ Recipient mice displayed a range of PCOS-like features including the appearance of insulin resistance, disrupted estrous cycles, increased numbers of cyst-like follicles and reduced corpora lutea populations.^24^ Collectively, these findings provide strong evidence to suggest that the gut microbiome plays an active role in PCOS pathogenesis. However, identification of the precise perturbations in gut microbiota that may contribute to the development of features of PCOS remains to be clearly defined and is a challenge as reports of alterations of the gut microbiota in PCOS women and animal models vary between studies for reasons not yet fully understood.^25^

Previously we revealed that the development of PCOS traits is influenced by dietary macronutrient balance.^26^ However, variations in dietary macronutrient balance had a minimal beneficial effect on ameliorating metabolic PCOS traits even though PCOS-like mice exhibited the same eating pattern and expended the same amount of energy compared to controls. These results suggest a difference in post-ingestive responses to diet between control and PCOS females, possibly food absorption and metabolism, which could potentially be mediated by the gut microbiome. Therefore, in the current study, we investigated the impact of diet on microbiota of a hyperandrogenic PCOS mouse model and identified changes related to both diet and PCOS (Experiment 1). We also examined if manipulating the gut microbiome using FMT from healthy controls could improve PCOS features (Experiment 2), which would indicate a potential role of the gut microbiome in mediating the development of PCOS traits.

## Results

### Effect of dietary macronutrient balance on microbial diversity and composition

**Figure 1.**
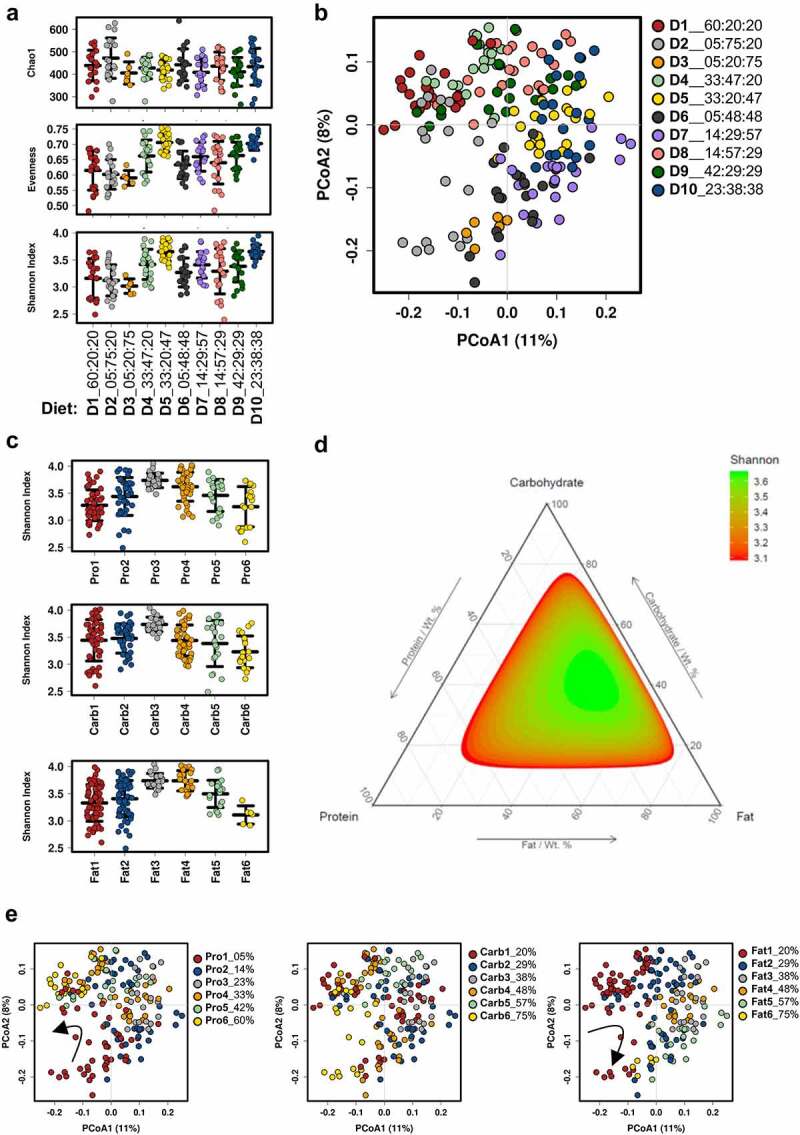
**Effect of dietary macronutrient balance on the gut microbiota of mice**. Control and PCOS-like mice were pooled for this analysis. (**A**) Measures of α-diversity: Chao1 species (OTU) richness, species evenness, and Shannon’s diversity index. Chao1 was not significantly different across diets (P = .14); however, species evenness (P < .0001) and Shannon’s diversity index (P < .0001) were significantly different across diets. Data are displayed as mean ± SEM and were analyzed by ANOVA (n = 6–20 mice/diet). X-axis denotes P:C:F ratio of each diet. (**B**) Principal coordinate analysis (PCoA) of the Bray-Curtis resemblance matrix generated from square-root transformed relative abundances at the OTU level (β-diversity). Proportion of variance explained by each principal coordinate (PCo) is denoted on the corresponding axis in parenthesis. Microbial composition was found to be significantly different (R^2^ = 0.25, P < .001) across diets using adonis (n = 6–20 mice/diet). (**C**) Measure of α-diversity indicated by Shannon’s diversity index with different macronutrient composition groups classified by color from 1–6 in ascending order for % protein, % carbohydrate and % fat content in diet (Table S2). Data indicate that all three macronutrients significantly impact gut microbial α-diversity (all P < .0001). Data are mean ± SEM and were analyzed by ANOVA (n = 6–60 mice/group). (**D**) Ternary plot of Shannon’s diversity index combining protein, carbohydrate and fat content showing a maximal peak of α-diversity at a specific ratio of protein, carbohydrate, and fat in the diet. **E**, PCoA of Bray-Curtis resemblance matrix (β-diversity) for dietary content of protein, carbohydrate, and fat. Microbial composition was found to be significantly different across protein groups (R^2^ = 0.15, P < .001), carbohydrate groups (R^2^ = 0.12, P < .001), and fat groups (R^2^ = 0.14, P < .001) using adonis (n = 6–60 mice per group). PCoA plots for the available amount of dietary protein and fat in the diet show that microbial β-diversity shifts gradually as each of these macronutrients increase in diet (arrows) .

To assess the overall influence of macronutrient balance in diet on the microbiota, data were pooled from control and PCOS-like mice within each of the 10 diet formulations, before a separate analysis of androgen-induced specific differences. Measures of microbial diversity (α-diversity) revealed that diet did not significantly affect the number of taxa present (Chao1 species (OTU) richness) in pooled control and PCOS-like mouse samples (Figure 1a). However, species evenness was significantly different across the 10 diets (P < .0001; Figure 1a). In turn, the Shannon’s diversity index was significantly affected by diet in pooled microbial samples of control and PCOS-like mice (P < .0001; Figure 1a). Furthermore, the mice allocated to experimental diet compositions 5 (P:C:F ratio 33:20:47) and 10 (P:C:F ratio 23:38:38) exhibited the highest α-diversity measurements of evenness and Shannon’s diversity index and thus had the highest impact on microbial α-diversity.

To compare microbial composition between diet groups, Bray-Curtis resemblance matrices (similarities) were calculated from the relative abundance of bacteria at the OTU level (β-diversity) and ordinated and tested using PCoA and adonis. These analyses revealed that microbial composition was significantly different between each diet type, as mice consuming the same diet clustered together but away from those consuming another diet type (adonis: R^2^ = 0.25, P < .001; Figure 1b).

We then investigated the influence of protein, carbohydrate, and fat on pooled control and PCOS-like samples. To elucidate if microbial diversity and composition, regardless of PCOS phenotype, are influenced by a specific macronutrient (protein, carbohydrate and/or fat), data were analyzed based on increasing energy availability from protein, carbohydrate, and fat across diets (Table S2). Analysis revealed that all three macronutrients (P, C and F) influenced microbial diversity as measured by Shannon’s diversity index (α-diversity) in control and PCOS-like mice pooled together (all P < .0001; Figure 1c), with the greatest diversity achieved on diets with medium amounts of energy from Pro (23%), Carb (38%) and Fat (38% and 48%). As peak α-diversity was observed in diets with medium percentage compositions of P, C and F (Figure 1c), a ternary plot was created to visualize if the individual macronutrient peaks coincided (Figure 1d). Results from the ternary plot confirm that in mice there is a maximal peak in Shannon’s diversity at a very specific ratio of P, C and F dietary content of 23% P, 38% C and 38% F (Figure 1d).

Bray-Curtis resemblance matrix (similarities) of the relative abundance of bacteria at the OTU level (β-diversity) was visualized using PCoA to compare individual macronutrient groupings (Table S2, Figure 1e). This analysis showed that microbial composition was significantly different across protein groups (R^2^ = 0.15, P < .001), carbohydrate groups (R^2^ = 0.12, P < .001), and fat groups (R^2^ = 0.14, P < .001), demonstrating the strong impact of macronutrients on the composition of the gut microbiota in mice. In particular, the amount of dietary energy pertaining to P and F exhibited a specific pattern in which the shift of β-microbial diversity changed progressively as dietary P and F increased (shown in change from red to yellow groups in Figure 1e). This shift in β-microbial diversity corresponding to P and F intake was observed to occur in opposite directions, as shown by arrows (Figure 1e).

### Effect of PCOS pathology on microbial diversity and microbial composition

**Figure 2.**
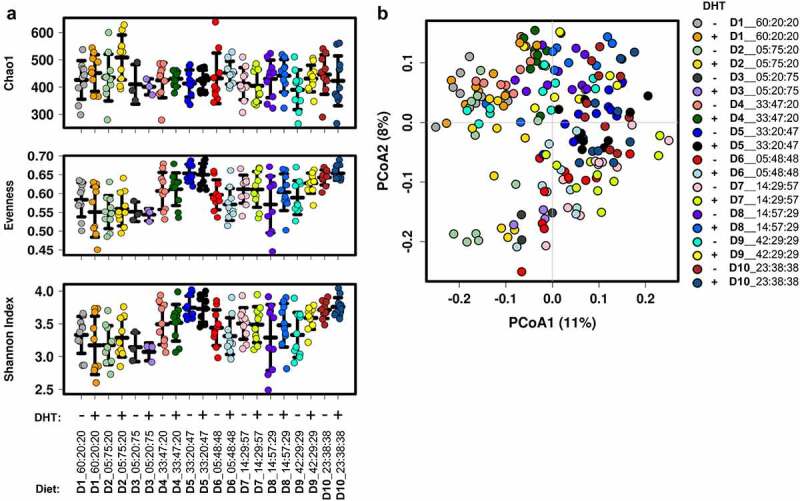
**Effect of dietary macronutrient balance on the gut microbiota of control (blank) and PCOS-like mice (DHT). (A**) Measures of α-diversity: Chao1, species evenness, and Shannon’s diversity index. No significant differences in α-diversity measures were observed between control and PCOS-like mice within each diet. Regardless of diet grouping, no differences in α-diversity were observed between control (blank) and PCOS-like (DHT) mice. Data are displayed as mean ± SEM and were analyzed by ANOVA (n = 3–10 mice/group). (**B)** Principal coordinate analysis (PCoA) of the Bray-Curtis resemblance matrix generated from square-root transformed relative abundances at the OTU level (β-diversity). Proportion of variance explained by each principal coordinate (PCo) is denoted in on the corresponding axis in parenthesis. The effect of DHT on the gut microbiota was tested using a range of models, showing PCOS pathology (DHT) had a significant effect on gut microbiota composition (n = 3–10 mice/group). Tested by PERMANOVA: Pseudo-F: 1.6, P = .017, df =172; adonis: R^2^ = 0.024, P =.005; Canonical correspondence analysis (CCA): F = 1.6, P = .002; and Redundancy analysis (RDA): F =1.4, P = .001.

To determine if there were androgen-induced differences in microbial diversity and composition between control and PCOS-like mice on each of the 10 experimental diet compositions, we analyzed the impact of diet on control and DHT-induced PCOS-like mice fecal samples separately. DHT treatment did not significantly change gut microbial α-diversity measurements of Chao1 species (OTU) richness, species evenness and Shannon’s diversity index between control and PCOS-like mice on the same diet (Figure 2a). This indicates that when ingesting the same diet, control and PCOS-like mice did not differ in their gut microbial intra-sample diversity. Moreover, a maximal microbial α-diversity, as indicated by Shannon’s diversity index, was observed in both control and PCOS-like mice on diet compositions 5 and 10 (Figure 2a; Table S1). Thus, excess androgen levels did not lead to additional changes in microbial diversity than those inflicted by diet alone.

In contrast, analysis of microbial composition, β-diversity, of control and PCOS-like mice across the ten diet formulations (Figure 2b) was found to be significantly influenced by the hyperandrogenic environment as tested by PERMANOVA (Pseudo-F: 1.6, P = .017, df = 172) and confirmed by CCA (F = 1.6, P = .002) and RDA (F = 1.4, P = .001) constrained analyses.

**Figure 3.**
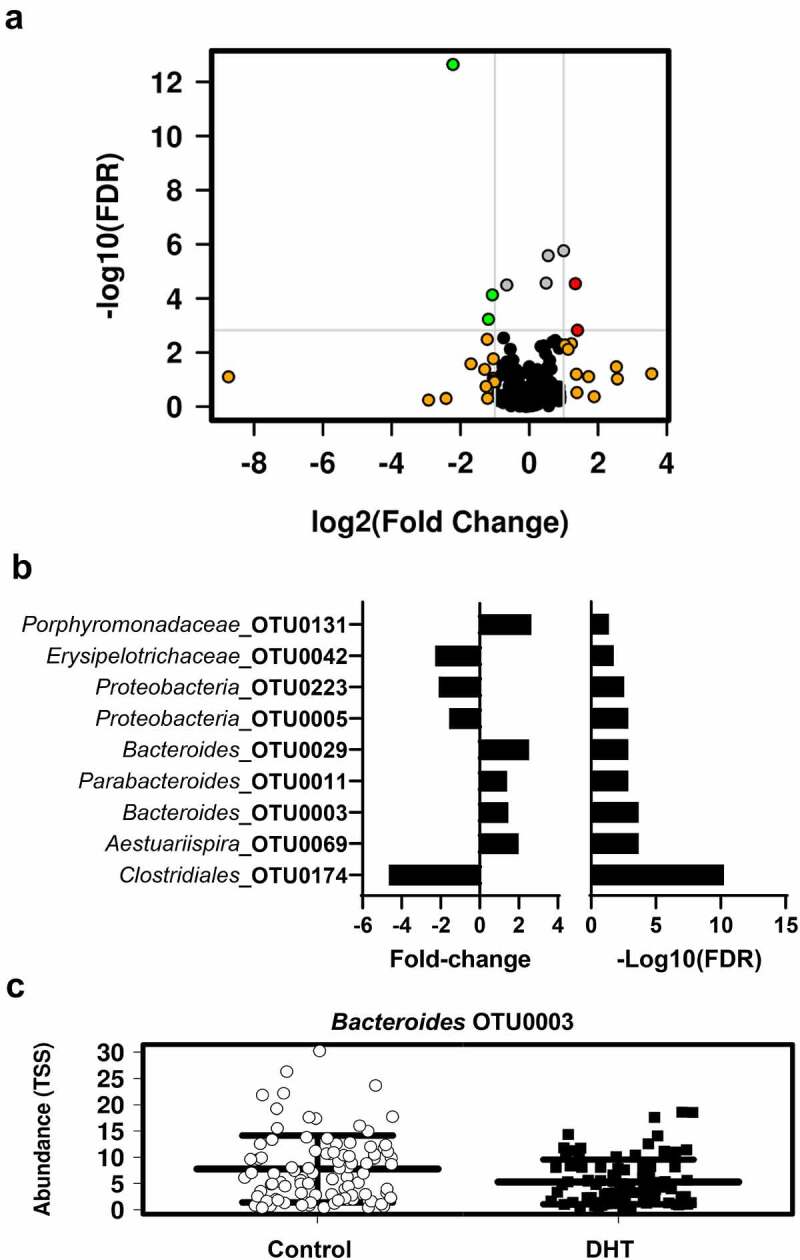
**Effect of DHT exposure on the relative abundance of bacterial taxa. (A)** Volcano plot of differentially abundant taxa between control and PCOS-like mice identified using DESeq2 analysis against the top 300 OTUs. The light gray horizontal line perpendicular to the y axis shows where P = .05 with points above the line having P < .05. The two light gray line perpendicular to the x axis shows fold-changes of 2 (log2 = 1) and −2 (log2 = −1). Green dots are OTUs with relative abundance significantly greater than two-fold in PCOS-like mice as compared to controls. Red dots are OTUs with relative abundance significantly greater than two-fold in controls as compared to PCOS-like mice. Grey dots are OTUs with relative abundance less than −2 or 2-fold significant difference between control and PCOS-like mice. Orange dots represent OTUs with relative abundances greater than two-fold difference between control and PCOS but were not significantly different. Black dots are OTUs with relative abundance less than −2 or 2-fold difference between control and PCOS but were not significantly different. (**B)** Graphical representation of fold-change values of the nine OTUs identified to have significantly different relative abundance between control and PCOS-like mice following false discovery rate (FDR) correction. (**C)** Scatterplots of the most abundant taxa out of the nine OTUs found to be significantly different between control and PCOS-like mice. Data are displayed as mean ± SEM (n = 93–94 mice/treatment group) .

Given that the androgen excess had an impact on global microbial composition, we went on to investigate which specific bacterial taxa were responsible for these differences. Using DESeq2, nine operational taxonomic units (OTUs) were identified to have significantly different relative abundances between control and PCOS-like mice (Figure 3a,b; Table S3), independent of diet. These differences ranged from – 4.6- to 2.6-fold (Table S3).

The most abundant taxa identified to be significantly different between control and PCOS-like mice was *Bacteroides* OTU0003 (Table S3). The relative abundance of *Bacteroides* OTU0003 was decreased in PCOS-like mice compared to control mice (control: 7.88% and PCOS 5.38%; fold change = 1.464; P < .0001; Figure 3c). The consensus sequences of *Bacteroides* OTU0003 was assessed using blastn against the NCBI database, which showed a 99.21% similarity to *Bacteroides acidifaciens*.

### PCOS-associated Bacteroides OTU0003 is differentially impacted by dietary macronutrient balance

**Figure 4.**
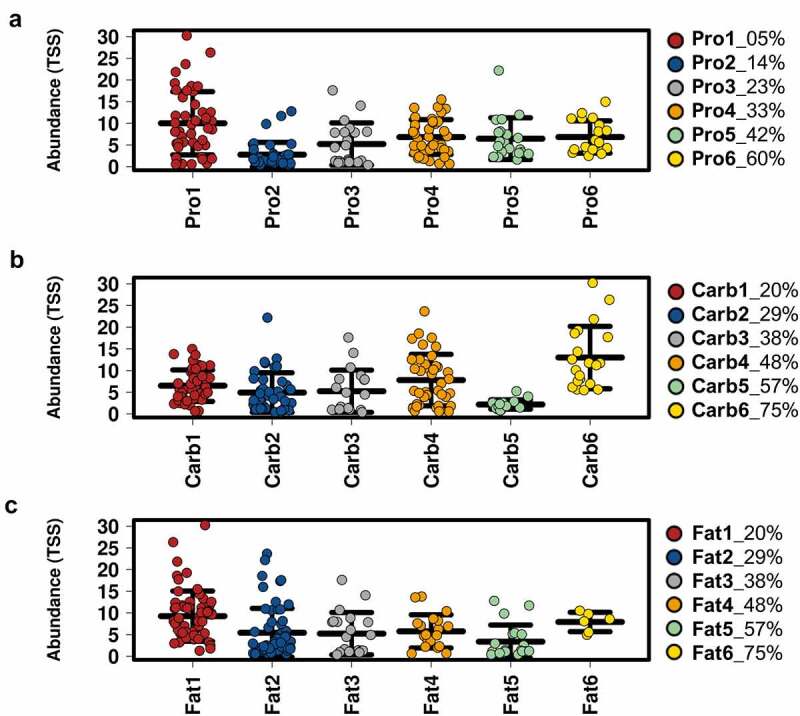
**Influence of protein, carbohydrate and fat in diet on *Bacteroides* OTU0003 abundance**. Relative abundance (%) of *Bacteroides* OTU0003 assessed against dietary content of protein (a), carbohydrate (b) and fat (c) in control and PCOS-like mice. Groups are classified for the amount of energy in the diet from 1–6 in ascending order for protein, carbohydrate and fat content (Table S2). Data are displayed as mean ± SEM (n = 6–60 mice per group). Relative abundance of *Bacteroides* OTU0003 is significantly altered across all three macronutrients as tested by ANOVA (all P < .0001). FDR corrected Pearson correlations revealed that Carb exhibited the greatest influence over the relative abundance of Bacteroides OTU0003 (Carb: r = 0.22, p = .0028, q = 0.015; Pro: r = −0.0091, p = .90, q = 0.94; Fat: r = −0.15, p = .040, q = 0.081) .

*B. acidifaciens* is reported to be inversely associated with obesity, with increased levels associated with prevention of the development of an obese phenotype in mice consuming a high fat diet.^27^ *Bacteroides* OTU0003 (*B. acidifaciens)* was the most abundant taxon, with a significant decrease in relative abundance in PCOS-like mice compared to controls (Figure 3c). As it was identified that diet had a stronger overall influence on gut microbiota α-diversity than DHT treatment, we investigated if dietary P, C and F had an influence on modulating *Bacteroides* OTU0003 in control and PCOS-like mice samples. Analysis revealed that the relative abundance of *Bacteroides* OTU0003 was significantly affected by the amount of P, C and F in the diet (Figure 4a-c; all P < .0001). *Bacteroides* OTU0003 was observed to have the highest relative abundance at low levels of P (5%) and F (20%) and high levels of C (75%) (Figure 4a-c). Interestingly, for dietary F, the relative abundance of *Bacteroides* OTU0003 decreased progressively as dietary F content increased, except for the highest dietary F % in diet (75%) in which a slight increase was observed (Figure 4c). However, further analysis using FDR corrected Pearson correlations against macronutrient intake uncovered that C was the macronutrient that had the strongest influence over the relative abundance of *Bacteroides* OTU0003 compared to P and F (C: r = 0.22, p = .0028, q = 0.015; P: r = −0.0091, p = .90, q = 0.94; F: r = −0.15, p = .040, q = 0.081).

### FMT did not ameliorate metabolic and reproductive PCOS features in PCOS-like mice

**Figure 5.**
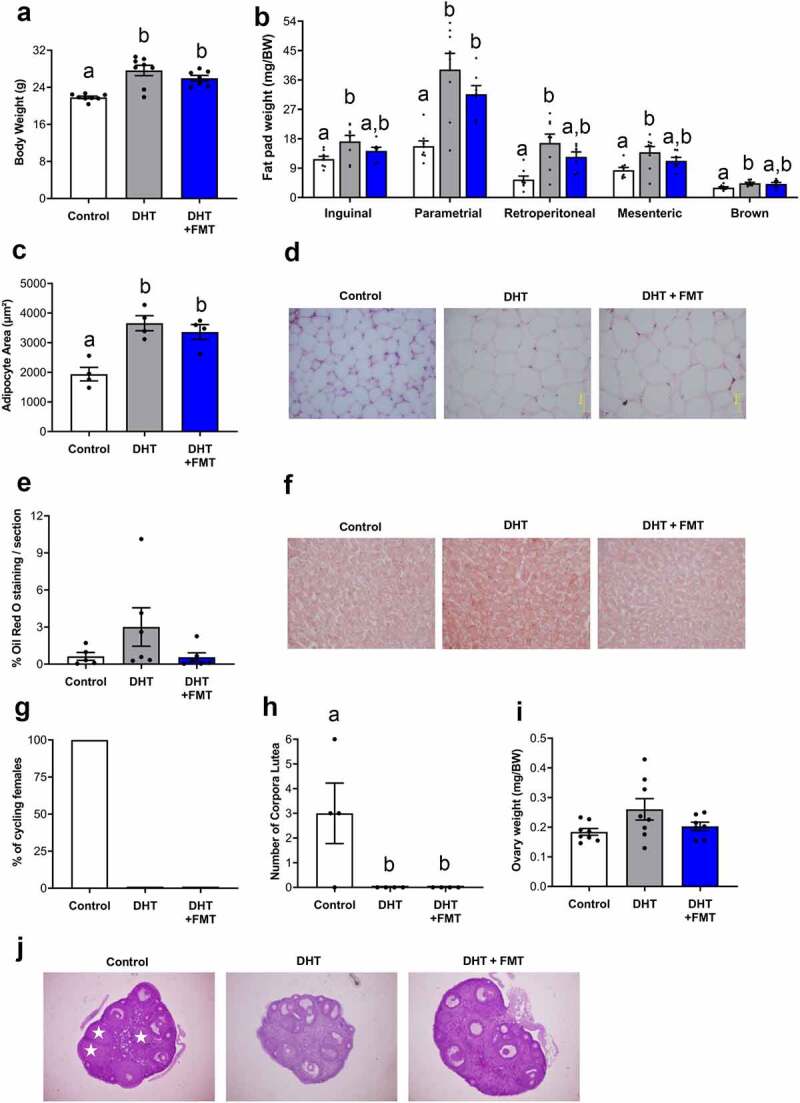
**FMT did not ameliorate metabolic and reproductive PCOS-like traits. (A**) Body weight, showing DHT treatment led to a significant increase in body weight compared to controls, which was not ameliorated by FMT. n =7-8/group. Data are the mean ± SEM. (**B**) Fat depot weights, showing PCOS-like mice displayed a significant increase in inguinal, parametrial, retroperitoneal, mesenteric and brown adipose tissue weights. However, FMT decreased inguinal, retroperitoneal, mesenteric and brown adipose tissue weights to weights comparable to control females. n = 7–8 mice/group. Data are the mean ± SEM. (**C**) Parametrial adipocyte area, showing that FMT treatment did not decrease adipocyte area in PCOS-like females. n = 4 mice/group. Data are the mean ± SEM. (**D**) Histological sections of representative parametrial fat pads from a control, a DHT and an FMT treated DHT mouse. Magnification 40x. (**E)** Percentage of oil red O liver lipid stain, showing no difference in lipid staining between all groups. n = 5–6 mice/group. Data are the mean ± SEM. (**F**) Histological sections of representative liver sections stained with oil red O. (**G)** Percentage of cycling females showing FMT did not restore estrous cyclicity in PCOS-like females. n = 7–8 mice/group. (**H**) Number of corpora lutea, showing FMT did not restore ovulation in PCOS-like females. n = 4 mice/group. Data are the mean ± SEM. **I**, Ovary weight, showing no significant difference in ovary weight between all groups. n = 7–8 mice/group. J, Histological sections of representative ovaries from a control, a DHT and an FMT treated DHT mouse. Data are the mean ± S.E.M.; different letters (‘a’ and/or ‘b’) denote significant statistical differences by one-way-ANOVA followed by *post hoc* comparisons with Tukey’s Multiple Comparisons Test (*P* <.05) .

The finding that diet had a stronger effect on the microbiome, yet DHT-induced PCOS caused some changes in bacterial population at the OTU level, prompt additional investigations into whether manipulating the gut microbiome could ameliorate PCOS-like traits by performing FMT from healthy mice into PCOS-like females. We hypothesized that healthy FMT would cause a shift in the gut microbiota of PCOS-like females and ameliorate PCOS features. Similar to previous studies,^28–30^ DHT treatment resulted in a significant increase in body weight (P < .001), inguinal (P < .05), parametrial (P < .001), retroperitoneal (P < .01), mesenteric (P < .05), and brown (P < .05) fat pad weights in PCOS-like mice, compared to placebo-treated control mice (Figure 5a and 5b). Body weight was unaffected by FMT-treatment in PCOS-like mice (Figure 5a). FMT-treated PCOS-like mice displayed comparable inguinal, parametrial, retroperitoneal, mesenteric, and brown fat pad weights to PCOS-like mice but were also statistically comparable (except parametrial fat) to weights observed in control mice (Figure 5b). DHT exposure led to a significant increase in the adipocyte area of parametrial fat depots compared to control females (P < .01), and FMT had no impact on this trait (Figure 5c and 5D, P < .01). Although a few PCOS-like mice exhibited an increase in the percentage of oil red O absorption, there was no significant difference in hepatic oil red O staining between the three groups (Figure 5e,f). Likewise, there was no significant difference in both serum fasting glucose levels and fed insulin levels between all three groups (Figure S2 A and B).

Estrous cycle irregularity is a key diagnostic criterion for PCOS, and the development of this trait was confirmed by the detection of estrous acyclicity in all PCOS-like mice (Figure 5g, p < .01). Similar to PCOS-like mice, 0% of FMT-treated PCOS-like mice displayed estrous cyclicity (Figure 5g). Oligo/anovulation is a hallmark reproductive characteristic of PCOS, and the development of this trait was confirmed by the detection of anovulation in PCOS-like females. Analysis of the ovarian histological section revealed that PCOS-like females displayed zero corpora lutea (CL) which was significantly different from controls that displayed an average of three CL/ovary (Figure 5h,j; p < .05). Similarly, CL were absent in the ovaries of all FMT-treated PCOS-like mice (Figure 5h,j). Ovary weight did not differ significantly between the three groups, although there was a tendency for ovarian weight to be increased in PCOS-like mice compared to control and FMT-treated PCOS-like mice (Figure 5i).

### FMT resulted in a significant shift of the PCOS gut microbiome composition toward that of the control gut microbiome

**Figure 6.**
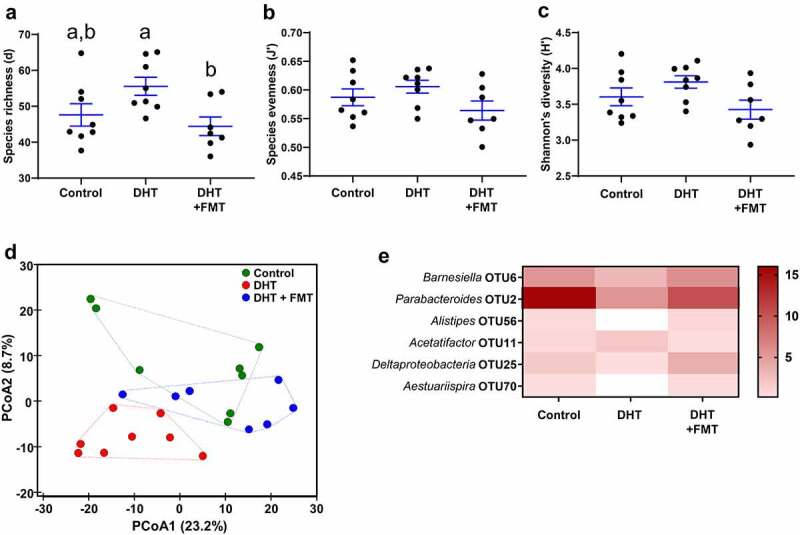
**FMT shift microbiota α and β-diversity of PCOS-like mice to profiles similar to controls**. α-diversity indicated by (a) species richness, showing FMT significantly decreased species richness in PCOS-like females compared to PCOS-like females not treated with FMT. Species evenness (b) and Shannon’s diversity index (c), showing no significant difference between all groups. n =7-8/group. Data are the mean ± SEM; different letters (‘a’ and/or ‘b’) denote significant statistical differences by one-way ANOVA followed by *post-hoc* comparisons with Tukey’s Multiple Comparisons Test (*P* <.05). (**D**) β-diversity indicated by principal coordinates analysis (PCoA) of Bray-Curtis resemblance matrix generated from square-root transformed relative abundances at the OTU level. Proportion of variance explained by each principal coordinate (PCo) is denoted on the corresponding axis in parenthesis. Microbial composition was found to be significantly different across groups (Pseudo-F: 1.7, P =.042, df =1) and body weight (Pseudo-F: 2.6, p =.003, df =1) using PERMANOVA. This significant difference across groups was confirmed using a one-way ANOSIM test (R =0.301, P =.002). n =7-8/group. (**E**) Heat map of the average relative abundances of the 6 key taxa between the 3 groups. In this graph, the OTUs are statistically significant (P <.05) and had an LDA score >3. The color of each cell from white to red corresponds to the relative abundance (normalized and log-transformed) of the OTUs from low to high. The genus level taxonomic assignment is shown on the left.

Measures of microbial α-diversity revealed that compared to control mice, there was no significant difference between the number of different taxa (species richness) in the gut microbiome of PCOS-like mice with or without FMT (Figure 6a-c). However, FMT into PCOS-like females resulted in a significant decrease in species richness compared to PCOS-like mice (Figure 6a, p < .05). Between all treatment groups there was no significant change in species evenness (Figure 6b) and Shannon’s diversity index (Figure 6c). Analysis of FMT species richness revealed FMT had a similar species richness to controls (Figure S3 A). To compare β-diversity, Bray-Curtis resemblance matrices (similarities) were calculated from the relative abundance of bacteria at the OTU level and plotted and tested using PCoA, PERMANOVA and ANOSIM to compare the microbial composition between the three groups. These analyses revealed that microbial composition was significantly different between control and PCOS-like mice, as mice in the same group clustered together and were distant to the other group (Figure 6d; PERMANOVA: Pseudo-F: 2.67, P = .003, df = 1,11; ANOSIM: R = 0.301, P = .002). This result validated our findings related to β-diversity in experiment 1, whereby DHT exposure had a significant effect on β-diversity in PCOS-like females (Figure 2b). FMT treatment of PCOS-like mice resulted in a shift in microbial composition (β-diversity) toward that of the control group (Figure 6d). Inclusion of FMT sample in Bray-Curtis resemblance matrices (similarities) revealed FMT had a similar microbial composition to controls (Figure S3 B).

LEfSe analysis identified six OTUs to be significantly different between control and PCOS-like mice and control and FMT-treated PCOS-like mice. FMT treatment resulted in a shift in the average relative abundances of the six key taxa in the PCOS gut microbiome toward that of the control (Figure 6e). Compared to control mice, in PCOS-like mice the relative abundance of *Barnesiella* OTU6 (P = .021), *Parabacteroides* OTU2 (P = .0023), *Alistipes* OTU56 (P = .0046), *Deltaproteobacteria* OTU25 (P = .016) and *Aestuariispira* OTU70 (P = .036) were significantly decreased, while the relative abundance of *Acetatifactor* OTU11 (P = .036) was significantly increased (Figure 6e). FMT treatment induced a shift in gut microbiome in PCOS-like mice, which was toward the gut microbiome of the control group. In PCOS-like mice, FMT treatment resulted in a significant increase in the relative abundance of *Barnesiella* OTU6 (P = .0012), *Parabacteroides* OTU2 (P = .011), *Alistipes* OTU56 (P = .0026), *Deltaproteobacteria* OTU25 (P = .0054) and *Aestuariispira* OTU70 (P = .015), but a significant decrease in the relative abundance of *Acetatifactor* OTU11 (P = .021) (Figure 6e).

## Discussion

The gut microbiota has emerged as a potential factor in the pathogenesis of PCOS.^31^ Both diet^32,33^ and a PCOS environment^12,20^ influence the gut microbiome, and the current study set out to define how dietary macronutrient balance and PCOS pathology interplay to influence gut microbiota. The results revealed that dietary macronutrient balance, has a stronger influence over changes in the gut microbiome than PCOS pathology. In spite of this, a hyperandrogenic PCOS environment itself still leads to changes in gut microbiota composition, and while healthy FMT treatment can shift the hyperandrogenic PCOS microbiome toward that of controls this was not sufficient to amend PCOS features.

Diet significantly affected microbial diversity and composition in all mice across the different diet compositions. Specifically, this study found that mice on the two diet compositions with the most balanced distribution of macronutrient content (33% P, 20% C, 48% F and 23% P, 38% C, 38% F), exhibited greatest α-diversity, which was reproduced through individual macronutrient analysis. Gut microbial composition (β-diversity) was very similar in mice on these two diets. In general, a high microbial diversity is considered to be beneficial for health.^34^ Interestingly, in this study, the identified macronutrient balance with greatest microbial diversity closely resembles that of a Mediterranean diet with a macronutrient energy distribution of 15–20% protein, 35–40% carbohydrate and 35–45% fat,^35^ which has been associated with numerous health benefits.^36^ Notably, in our previous study, we also observed that a dietary macronutrient ratio similar to a Mediterranean diet was able to rescue ovulatory dysfunction in this PCOS mouse model. Taken together, these results suggest clinical studies assessing the effects of a diet with a macronutrient balance similar to that of a Mediterranean diet would be of particular interest in women with PCOS.

Lowest microbial diversity and changes in microbial composition were observed in mice on diets that contained the highest content of protein, carbohydrate or fat. This result is congruent with a previous study that investigated the influence of macronutrient balance in mice fed one of 30 diets in which microbial diversity decreased as protein and carbohydrate consumption increased.^37^ This decrease in α-diversity is also in line with studies in mice fed either a high fat diet^38–40^ or high carbohydrate diet,^40^ where a decrease in gut microbial diversity has been reported. In humans, reduced carbohydrate (polysaccharide) intake is reported to lead to a decrease in polysaccharide-utilizing gut bacteria, which consequently leads to an overall decrease in microbial diversity,^41,42^ consistent with our observed decrease in microbial diversity on a high protein, low carbohydrate diet. Together these studies support this study’s findings that diet exerts a major influence over the gut microbiome, therefore dietary interventions have the potential to serve as treatment options for various metabolic conditions, such as PCOS.

In this study, gut microbial α-diversity was not influenced by PCOS pathology as PCOS-like and control mice ingesting the same diet displayed no difference in microbial diversity. This result is in line with a study that observed no change in α-diversity between control and a prenatal PCOS mouse model exposed to a high fat/high sugar diet.^19^ Likewise, a recent human study using whole genome shotgun sequencing revealed no significant difference in gut bacterial α-diversity between patients with and without PCOS.^24^ However, other rodent studies^17,18,20^ and some clinical studies^12–14^ have reported lower α-diversity in women suffering from PCOS compared to healthy controls. These differences are potentially due to variations in PCOS phenotypes, but also variations in diet. Overall, the findings from this study indicate that diet composition has a stronger impact in determining gut microbial α-diversity, beyond the hyperandrogenic PCOS environment.

On the other hand, microbial composition was found to be significantly different between control and PCOS-like mice. Several studies have reported altered microbial composition in PCOS rodent models^17,18,20,23^ and women with PCOS.^13,14,24^ One potential explanation for this shift is the presence of elevated levels of androgens, as a study in women with PCOS identified that β-diversity was negatively correlated with hyperandrogenism.^13^ Moreover, studies in humans and rodents support the notion of sex steroids influencing the gut microbiome.^43,44^ The mechanism by which androgens alter microbial diversity and affect PCOS is not understood, but there are reports of high levels of androgen in feces of mice and men^45^ and the presence of androgen receptors in the large intestine.^46^ We previously reported that serum testosterone levels are comparable between control and DHT-induced PCOS-like mice.^26,47,48^ Similar findings were evident in the cohort of mice used for experiment 1 (diet intervention).^26^ Since DHT, unlike testosterone, is not able to be aromatized into estradiol, this finding suggests that androgens acting via the androgen receptor may play a direct role in the interaction between gut microbe and host interactions.

*Bacteroides* OTU0003 was significantly decreased in PCOS-like mice. Similar studies on PCOS rodent models have also reported a decrease in *Bacteroides*.^17,18,20^ Interestingly, *Bacteroides* OTU0003 shares 99.21% similarity to *Bacteroides acidifaciens*, treatment with *B. acidifaciens* has been reported to prevent the development of obesity and improve insulin sensitivity, and energy metabolism in mice fed a high fat diet.^27^ This aligns with findings from our previous study^26^ where these mice displayed an overall sensitivity to caloric intake and were generally overweight compared to controls. The relative abundance of *Bacteroides* OTU0003 in mice was significantly influenced by the amount of carbohydrates in their diet, with the highest relative abundance achieved on diets with high levels of carbohydrate (75%). Accordingly, previous studies in mice showed that consuming a diet high in fiber^49^ or specifically incorporating mannan-oligosaccharide into the diet^50^ increased the prevalence of *Bacteroides acidifaciens*. One source of dietary carbohydrates in this study was dextrinized starch, a form of complex carbohydrate that is mostly digested in the colon, therefore we hypothesized that fiber is potentially the modulator of *Bacteroides* OTU0003 relative abundance. Given that diet was observed to have a stronger effect on gut microbiota than PCOS pathology, the data suggests that the focus in PCOS should be toward ingestion of diets with increased fiber as prebiotic support for maintaining a population of healthy bacteria, which could be further investigated in clinical studies.

FMT treatment of healthy control feces in PCOS-like mice significantly lowered α-diversity and shifted β-diversity of the gut microbiome toward that of the control mice. These results are congruent with a previous study that reported the restoration of normal, healthy gut microbiome composition after FMT treatment from healthy control mice in a letrozole-induced PCOS mouse model.^16^ This suggests that FMT treatment has a beneficial effect in driving microbial composition. It should be noted that in this experiment PCOS-like mice did exhibit a trend toward increased microbial community richness compared to controls, unlike in experiment 1, which is likely due to a difference in the baseline gut microbiota given that these experiments were conducted at different times, which is a limitation of this study. In this study, control and PCOS-like mice displayed differentially abundant taxa, with the most abundantly different bacteria being *Barnesiella* OTU6 (<90% similarity to known species) and *Parabacteroides* OTU2 (100% similarity to *Parabacteroides goldsteinii*), which were significantly decreased in PCOS-like mice compared to controls. Both *Barnesiella* and *Parabacteroides* species have been suggested to confer beneficial impacts on host metabolism. One study revealed that oral treatment of live *P. golsteinii* bacteria in obese mice resulted in improvements in intestinal integrity and reduction in weight gain, insulin resistance and inflammation.^51^ Altogether, these results reveal that FMT treatment can lead to a significant shift in the PCOS gut microbiome.

Although we observed a shift in gut microbial composition, FMT treatment did not significantly impact PCOS reproductive and metabolic features. Still, a possible decrease in overall adiposity in PCOS-like mice may be evident, as FMT-treated PCOS-like mice exhibited inguinal, mesenteric, retroperitoneal and brown fat depot weights comparable to those of control mice. Furthermore, fasting glucose levels and fed insulin levels were unaffected by DHT or DHT+FMT treatment, consistent with our previous studies. These results differ from previously published studies in which exposure to a healthy gut microbiome resulted in both reduced weight gain and abdominal adiposity,^23^ as well as improved estrous cyclicity and/or ovulation,^16,23^ in a letrozole-induced PCOS mouse model. This inconsistency may be due to a difference in PCOS mouse models, mode of exposure to a healthy gut microbiome and/or length of time of intervention resulting in variation in PCOS phenotypes. Specifically, it should be noted that the sustained supraphysiological levels of DHT used to develop the PCOS mouse model with a strong metabolic phenotype may slow full recovery of the PCOS phenotype, which is a limitation of this study.

Altogether, these findings demonstrate that diet exerts a stronger influence over the gut microbiome than PCOS pathology. The hyperandrogenic PCOS environment does change the gut microbiota β-diversity and is associated with specific decreases in the *Bacteroides* species associated with metabolic health in PCOS-like mice. In summary, hyperandrogenic PCOS does not appear to change the intestinal microbiome’s response to dietary protein, carbohydrate and fat, but instead altered the relative abundance of specific gut microbiota species that could potentially hinder healthy metabolic functions. Collectively, these findings support further research into the mechanism by which specific gut microbes can influence the development of PCOS features and if these vary with different PCOS phenotypes.

## Materials and methods

### Mice

#### Experiment 1

Assessment of gut microbiota of control and PCOS-like mice exposed to 1 of 10 different diets. Control and PCOS-like mouse fecal samples were analyzed from our previously published studies.^26^ Briefly, 3- to 4-week-old C57Bl/6 J female mice were housed in groups of 2 or 3 mice per cage (4–5 cages per diet) and maintained under standard housing conditions (ad libitum access to food and water in a temperature-controlled and humidity-controlled, 12-h light/dark environment) at the ANZAC Research Institute. At 7 weeks of age, control and dihydrotestosterone (DHT)-induced PCOS-like mice were switched from the standard chow diet and provided ad libitum access to one of the 10 experimental diets varying in protein, carbohydrate, and fat content (Specialty Feeds) (Figure S1 and Table S1). The selection of macronutrient content used in the experimental diets was chosen, based on our previous publications.^52,53^

#### Experiment 2

Impact of healthy FMT in PCOS-like mice. C57BL/6 J female mice were purchased at 3-weeks of age. The mice were maintained under standard housing conditions (ad libitum access to food and water in a temperature- and humidity-controlled, 12-hour light/12-hour dark environment) at the Biological Resources Center facility at UNSW (Sydney, Australia). The mice were divided into three groups, each with 7–8 mice: Control + saline, DHT + saline, and DHT + antibiotic + FMT (Figure S1). All three groups of mice were fed the Soya Oil Modification Ain93G diet (Specialty Feeds). All surgical procedures were performed under isoflurane inhalation. All procedures were approved by the UNSW Animal Care and Ethics Committee (19/17b).

### Generation of PCOS mouse model

PCOS-like mice were generated by postnatal exposure to DHT. Female mice were implanted subcutaneously with either a blank or a DHT-filled implant at 3–4 weeks of age. Implants were made using a 1-cm SILASTIC brand tubing with an inner diameter of 1.47 mm and an outer diameter of 1.95 mm made of medical grade–grade silicon (Dow Corning Corp, catalog no. 508–006). Implants were filled with ~10 mg DHT before being sealed. DHT-filled implants provide a continuous release of DHT over 90 days and induce a PCOS-like phenotype in mice.^29,30^ This PCOS mouse model was chosen for this study to reproduce both metabolic and reproductive features of PCOS, which more closely resembles the ‘obese’ PCOS phenotype that affects most women with PCOS. Blank implants were empty 1-cm sized SILASTIC tubing.

### Fecal sample collection, processing, and transplantation

Experiment 2. At 10 weeks of age, fecal samples were collected from all 8 control mice once a week for 6 weeks for FMT-processing. The fresh fecal pellets were resuspended in saline/Phosphate Buffered Solution in a 1.5 ml Eppendorf® tube, then vortexed until homogenized. It was then centrifuged at low speed (~200 x *g*). The resulting supernatant was collected and added to sterile 10% glycerol saline solution. All the samples were frozen immediately after collection and stored at – 80°C. Once all 6 weeks of sample supernatants were stored, they were all thawed once to pool all samples together, aliquoted into daily use tubes and frozen for later use. At 17-weeks of age, placebo gavage (10% glycerol saline solution) was administered to all control mice and 8 PCOS-like mice, while antibiotics were given to another 7 PCOS-like mice, 3 times/week for 1 week, to deplete their gut microbiota. At 18-weeks of age, 3 times/week for 2 weeks, placebo gavage was administered to all control (Control + saline group), and PCOS-like mice (DHT + saline group), while the PCOS-like mice previously treated with antibiotics were treated with pooled fecal supernatant (DHT + antibiotic + FMT group).

### Gut microbiome sample collection

At the end of the experiment, the entire intestinal tract was removed from mice from both experiments 1 and 2, and fecal contents from the colon were collected aseptically. All samples were snap frozen in liquid nitrogen and stored at – 80°C immediately after collection until DNA extraction.

### Fecal sample handling and sequencing

Fecal samples were homogenized, and DNA extracted using QIAmp ® PowerFecal ® DNA Kit (Hilden, Germany) following the manufacturer’s instructions. DNA concentration was measured using a NanoDrop 1000 Spectrophotometer (ThermoFisher).

Sequencing procedures were performed at the Ramaciotti Center for Genomics (UNSW Sydney). DNA was used in a PCR reaction to amplify the V4 region of the bacterial 16S rRNA gene using the following primers:

515 f (AATGATACGGCGACCACCGAGATCTACAC XXXXXXXX TATGGTAATT GT GTGYCAGCMGCCGCGGTAA) and

806 r (CAAGCAGAAGACGGCATACGAGAT XXXXXXXXXXXX AGTCAGCCAG CC GGACTACNVGGGTWTCTAAT) and the Platinum Hot Start PCR Master Mix (2x) from ThermoFisher (cat.no. 17000–11). The thermocycler conditions for amplification were activation at 94°C for 3 min followed by 35 cycles of amplification that included denaturation at 94°C for 45 seconds, annealing at 50°C for 1 minute, extension at 72°C for 1 minute and 30 seconds, and one final extension cycle at 72°C for 10 minutes; finally, the samples were cooled and held at 4°C. Amplified samples were then visualized on a 2% e-gel with an expected band size for 515 F/806 R of approximately 300–350 bp. PCR products were then cleaned and normalized using SequalPrep Normalization plates. Normalization was performed according to the manufacturer’s instructions. PCR products were then cleaned using beads on a portion of the pooled library, which therefore concentrated on it and allowed for the removal of any residual small fragments present. To ensure accurate sizing information, the clean amplicon pooled products were run on a 2200 TapeStation (or Agilent Bioanalyzer or LabChip GXII). Then, the concentration of the final clean pool was measured using a qubit. Finally, amplicon sequencing was performed with the Illumina MiSeq Reagent kit v3 (2x250 base pairs) .^54^

### Raw read analysis

Raw reads were analyzed using the MiSeq standard operating procedures within Mothur v1.39.1^54,55^ with the SILVA database and Ribosomal Database Project reference employed for alignment and classification, respectively. Quality filtering of 16S rRNA sequences and the subsequent subsampling of the data resulted in n = 21,361 total clean reads/sample for experiment 1 and n = 16,976 total clean reads/sample for experiment 2.

### Analysis of microbial communities

Microbial communities were examined for α-diversity and β-diversity, and changes in individual taxa. All tests were performed in Primer-E v6, Calypso,^56^ GraphPad Prism v7, and R. Before diversity comparisons, the operational taxonomic unit (OTU) counts were normalized by a total sum (% relative abundance) followed by square-root transformation. Chao1 species (OTU) richness, species evenness and Shannon diversity index were used as α-diversity measures, and linear mixed models were employed to examine the effects of DHT, FMT, diet and macronutrient composition. To assess the effect of each individual diet on microbial diversity measurements, diet groupings in Table S1 were used as depicted in Figure 1a-b and Figure 2a-b utilized. To assess the direct effect of each individual macronutrient (protein, carbohydrate, and fat), data were grouped by the percentage of P (5% to 60%), C (20% to 75%) and F (20% to 75%) present in each diet (Table S1), producing six different groups for each macronutrient (Table S2). Classification of diets into these six different groups, ranging from low to high concentrations of each macronutrient, allowed detailed analysis of the influence of each macronutrient based on its availability. Figure 1c-d and 1f and Figure 4a-c were analyzed using macronutrient classifications in Table S2. The ternary plot was generated using the R packages ggtern and ggplot2.^57^ For β-diversity comparisons, the Bray-Curtis resemblance matrix was generated from the square-root transformed relative abundances of OTU counts and visualized through principal coordinate analysis (PCoA). Adonis, PERMANOVA, Canonical correspondence analysis (CCA), and redundancy analysis (RDA) were used to examine the effects of diet, macronutrient composition and DHT implant using Calypso or Primer-E v6. Identification of differentially abundant taxa was performed using DESeq2.^58^

### Assessment of estrous cyclicity

Estrous-cycle stage was analyzed using vaginal epithelial cell smears taken daily for 5 weeks prior to collection.^29,59,60^ Smears were collected using 15 μL of 0.9% sterile saline and transferred to glass slides to air dry. Dry smears were stained with 0.5% toluidine blue before examination under a light microscope. Estrous-cycle stage was determined based on the presence or absence of leukocytes, cornified epithelial cells, and nucleated epithelial cells. Proestrus was characterized by the presence of mainly nucleated and some cornified epithelial cells; estrous by the presence of mostly cornified epithelial cells; metestrus by the presence of both cornified epithelial cells and leukocytes; and diestrus by the presence of predominantly leukocytes.

### Ovary preparation and morphological analysis

Ovaries were dissected, weighed, and fixed in 4% (weight/vol) paraformaldehyde overnight at 4°C and stored in 70% (vol/vol) ethanol before histological processing and analysis. Four Ovaries per group were processed through graded alcohol and embedded in glycol methacrylate resin (Technovit 7100; Heraeus Kulzer). Embedded ovaries were sectioned at 20 μm, stained with periodic acid-Schiff and counterstained with hematoxylin. The corpora lutea population was quantified as previously described,^29,59,60^ where whole-section images of every third section were taken under a light microscope using a DP70 Olympus camera and corpora lutea was identified with morphological properties consistent with luteinized follicles and observed through several serial sections.

### Adipose tissue analysis

Parametrial fat pads were weighed, fixed in 4% paraformaldehyde, embedded in paraffin, and sectioned at 8 μm. Sections were stained with hematoxylin and eosin and imaged at 40x magnification under the Olympus BX60 light microscope for histomorphometric analysis. Five different pictures were taken and assessed from each of three sections of the fat pad, with a minimum of 200 μm separating these sections. Adipocyte area was quantified using ImageJ version 1.51 software (NIH), as previously described.^29,59^

### Assessment of hepatic steatosis

Livers were first weighed whole before excision of the right lateral lobe. The right lateral lobe was fixed in 10% neutral-buffered formalin overnight at 4°C and then soaked in 30% sucrose (weight/vol) for 48 h; 1-cm × 1-cm portion of the fixed right lateral lobe was excised and embedded in an OCT compound (Tissue-Tek). 10-μm sections were cryo-sectioned from the embedded liver portion and air-dried onto slides. To visualize lipid deposition, slides were stained with oil red O in 60% isopropanol,^48,60^ before histo-morphometric analysis. ImageJ version software (NIH) was used to quantify oil red O-positive staining. Three separate images were analyzed from three different sections taken 200 μm apart.

### Statistical analyses of reproductive and metabolic PCOS traits

Statistical analyses were performed using GraphPad Prism v9. The normal distribution of the data was calculated using the Shapiro–Wilk test. Data that were not normally distributed were transformed before analysis using square-root or log transformation. Data that were not normally distributed after transformation were analyzed using the non-parametric Kruskal–Wallis test followed by Dunn’s multiple comparison test as a *post hoc* test. Data that were normally distributed were analyzed using one-way ANOVA followed by Tukey’s multiple comparison test as a *post hoc* test. P values <.05 were considered statistically significant.

## Supplementary Material

Supplemental Material

## Data Availability

Data sets generated are available in the European Nucleotide Archive repository under the accession number PRJEB48536.
