# Supplementary material for: The interplay between PCOS pathology and diet on gut microbiota in a mouse model
Source: Gut Microbes. 2022 Jul 4;14(1):2085961. doi: 10.1080/19490976.2022.2085961 (PMC9450977; doi:10.1080/19490976.2022.2085961)
Supplement: Supplemental Material [file KGMI_A_2085961_SM3385.pdf]

Supplementary Information

The interplay between PCOS pathology and diet on gut microbiota in a mouse model

Valentina Rodriguez Paris[1], Xin Yi Denise Wong[1], Samantha M Solon-Biet[2], Melissa C Edwards[1,3], Ali Aflatounian[1], Robert B Gilchrist[1], Stephen J Simpson[2], David J Handelsman[3], Nadeem O Kaakoush[4], Kirsty A Walters[1,3]

[1]Fertility & Research Centre, School of Clinical Medicine, University of New South Wales Sydney, NSW 2052, Australia.
[2]Charles Perkins Centre, University of Sydney, Sydney, NSW 2006, Australia
[3]ANZAC Research Institute, University of Sydney, Sydney, NSW 2139, Australia
[4]School of Medical Sciences, University of New South Wales Sydney, NSW 2052, Australia

Author for correspondence
Dr Valentina Rodriguez Paris, Fertility & Research Centre, School of Women's and Children's Health, University of New South Wales Sydney, NSW 2052, Australia. Email: v.rodriguezparis@unsw.edu.au

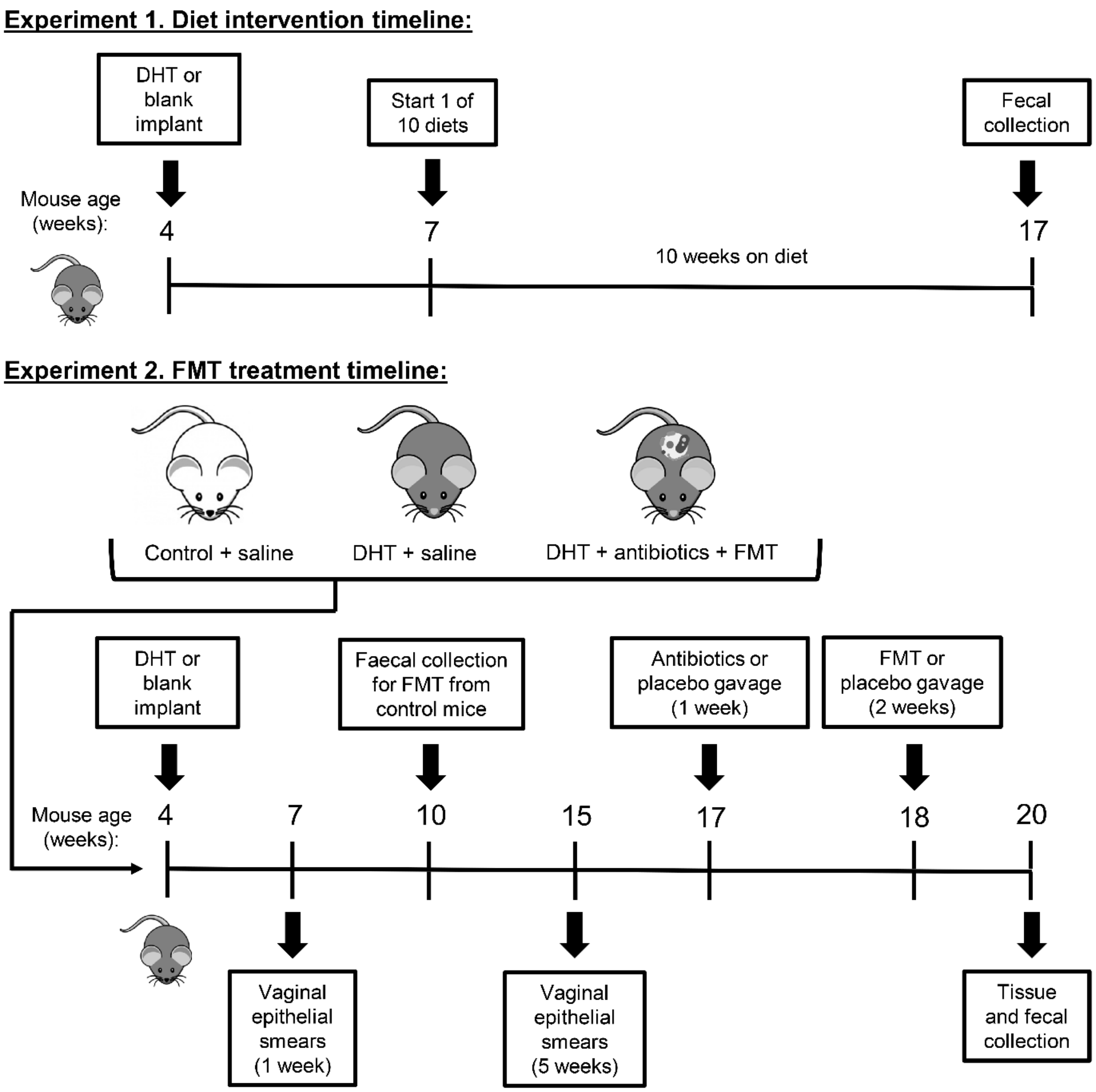

Figure S1. Experimental design. Experiment 1: PCOS was induced in female mice by s.c. insertion of a dihydrotestosterone (DHT) implant in peripubertal mice. Control mice were implanted with a blank (empty) pellet. At 7 weeks of age, control and PCOS mice were allocated to 1 of 10 diets for 10 weeks. Fecal pellets were collected after dietary intervention when mice were 17 weeks old. Experiment 2: PCOS was likewise induced in peripubertal mice by DHT implant insertion and control mice were implanted with a blank implant. Collection of fecal matter from control mice was performed prior to experimental treatment. After 12 weeks of androgen exposure, a subset of PCOS-like mice were treated via oral gavage with antibiotics 3 times per week for 1 week, followed by FMT treatment 3 times per week for 2 weeks, while control and the other subset of DHT mice were treated via oral gavage with placebo/saline 3 times per week for 3 weeks. Estrous cycling was assessed before the start of antibiotic treatment for 5 weeks. Serum, tissues and fecal pellets were collected after FMT or placebo treatment when mice were 20 weeks old. Body weight was recorded weekly for the duration of the experiment.

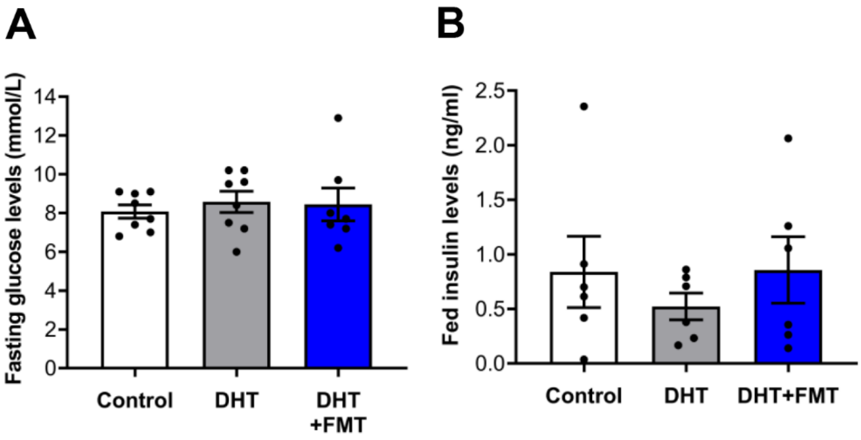

Figure S2. **Markers of insulin sensitivity. A**, Fasting glucose levels showing no significant difference between control, DHT and DHT+FMT groups. **B**, Fed insulin levels showing no significant difference between control, DHT and DHT+FMT groups. Data are the mean ± S.E.M.; statistical differences tested by one-way (*P*<0.05).

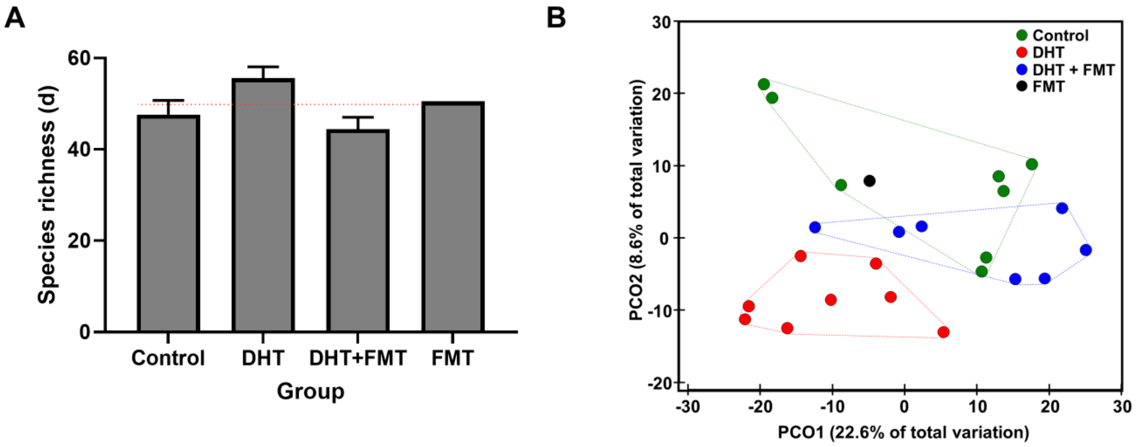

Figure S3. FMT treatment displays similar gut microbial α and β-diversity to controls. **A**, Species richness of control, DHT, DHT+FMT and FMT, showing FMT treatment species richness was similar to controls. **B**, β-diversity indicated by principal coordinates analysis (PCoA) of Bray-Curtis resemblance matrix, showing FMT treatment (black dot) had a similar microbial composition to controls (green dots), thus explaining the shift in microbial composition towards that of control female mice.

Diet	1	2	3	4	5	6	7	8	9	10
%P	60	5	5	33	33	5	14	14	42	23
%C	20	75	20	47	20	48	29	57	29	38
%F	20	20	75	20	47	48	57	29	29	38

Table S1. Macronutrient compositions of the 10 experimental diets.
The percentage protein (P), carbohydrate (C) and fat (F) were calculated as a percentage of the total energy for each diet. The sources for protein were casein and methionine, for carbohydrate sucrose, wheat starch and dextrinized cornstarch and for fat soya bean oil. Other ingredients, such as cellulose, were kept to similar proportions. All diets were supplemented with a vitamin mix (vitamin A, D3, E, K, C, B1, B2, Niacin, B6, pantothenic acid, biotin, folic acid, inositol, B12 and choline) and mineral mix (Ca, P, Mg, Na, C, K, S, Fe, Cu, I, Mn, Co, Zn, Mo, Se, Cd, Cr, Li, B, Ni and V) to levels found in AIN-93G (control diet formulation developed by the American Institute of Nutrition for optimal growth, pregnancy and lactation in rodents).

	Pro1	Pro2	Pro3	Pro4	Pro5	Pro6
%	5	14	23	33	42	60

	Carb1	Carb2	Carb3	Carb4	Carb5	Carb6
%	20	29	38	48	57	75

	Fat1	Fat2	Fat3	Fat4	Fat5	Fat6
%	20	29	38	48	57	75

Table S2. Macronutrient percentages used to group experimental diets.
Protein, carbohydrate and fat grouping, based on % of each macronutrient in each diet, used for analysis in Fig. 5.3, 5.4 and 5.9. Across the 10 experimental diets, energy intake ranged from 5% to 60% protein (Pro), 20% to 75% carbohydrate (Carb) and 20% to 75% fat (Fat).

Taxa	P-value	Q (FDR)	Blank	DHT	Fold Change
**p_*Firmicutes*_o_*Clostridiales*_OTU0174**	2.3E-13	5.7E-11	0.0084	0.039	-4.655
**p_*Proteobacteria*_g_*Aestuariispira*_OTU0069**	0.0000017	0.00022	0.24	0.12	1.999
**p_*Bacteroidetes*_g_*Bacteroides*_OTU0003**	0.0000026	0.00022	7.88	5.38	1.464
**p_*Bacteroidetes*_g_*Parabacteroides*_OTU0011**	0.000027	0.0013	2.5	1.78	1.405
**p_*Bacteroidetes*_g_*Bacteroides*_OTU0029**	0.000029	0.0013	1.21	0.48	2.532
**p_*Proteobacteria*_c_OTU0005**	0.000032	0.0013	2.85	4.48	-1.574
**p_*Proteobacteria*_c_OTU0223**	0.000074	0.0027	0.0078	0.016	-2.104
**p_*Firmicutes*_f_*Erysipelotrichaceae*_OTU0042**	0.00059	0.018	0.28	0.65	-2.277
**p_*Bacteroidetes*_f_*Porphyromonadaceae*_OTU0131**	0.0015	0.042	0.066	0.025	2.64

Table S3. The nine most abundant OTU's identified as being significantly different between control (blank) and PCOS (DHT) mice. DESeq2 analysis identification of 9 OTUs found to be significantly different between control and PCOS-like mice after false discovery rate (FDR) correction.
